# Parietal thickness predicts middle temporal area (V5) motion responses in 7-year-old children born very preterm

**DOI:** 10.1093/cercor/bhag089

**Published:** 2026-06-24

**Authors:** Linda Nguyen, Andrew E Silva, Tanya Poppe, Myra Leung, Jane M Alsweiler, Joanna Black, Jane E Harding, Anna C Tottman, Benjamin Thompson, PIANO Study Group, Frank H Bloomfield, Janene B Biggs, Coila Bevan, Kelly Fredell, Sabine Huth, Christine Kevan, Geraint Phillips, Jennifer A Rogers, Heather Stewart, Kathryn A Williamson, Trecia A Wouldes, Yannan Yiang

**Affiliations:** School of Optometry and Vision Science, University of Waterloo, 200 Columbia St W, Waterloo, ON N2L 3G1, Canada; School of Optometry and Vision Science, University of Waterloo, 200 Columbia St W, Waterloo, ON N2L 3G1, Canada; School of Optometry and Vision Science, University of Auckland, 85 Park Road, Grafton, Auckland 1023, New Zealand; School of Optometry and Vision Science, University of Auckland, 85 Park Road, Grafton, Auckland 1023, New Zealand; Discipline of Optometry and Vision Science, University of Canberra, 11 Kirinari Street, Canberra, ACT 2617, Australia; Department of Paediatrics: Child and Youth Health, University of Auckland, Private Bag 92019, Auckland 1142, New Zealand; Newborn Services, National Women’s Health, Auckland City Hospital, 2 Park Road, Grafton, Auckland 1010, New Zealand; School of Optometry and Vision Science, University of Auckland, 85 Park Road, Grafton, Auckland 1023, New Zealand; Liggins Institute, University of Auckland, 85 Park Rd, Grafton, Auckland 1023, New Zealand; Liggins Institute, University of Auckland, 85 Park Rd, Grafton, Auckland 1023, New Zealand; Neonatal Services, Royal Women’s Hospital, 20 Flemington Road, Parkville, Melbourne, VIC 3052, Australia; School of Optometry and Vision Science, University of Waterloo, 200 Columbia St W, Waterloo, ON N2L 3G1, Canada; School of Optometry and Vision Science, University of Auckland, 85 Park Road, Grafton, Auckland 1023, New Zealand; Liggins Institute, University of Auckland, 85 Park Rd, Grafton, Auckland 1023, New Zealand; Centre for Eye and Vision Research, 17W Hong Kong Science Park, Pak Shek Kok, Shatin, Hong Kong

**Keywords:** dorsal stream, functional magnetic resonance imaging, global motion processing

## Abstract

Very preterm birth has been associated with altered brain development and impaired global motion processing within the dorsal visual stream. We explored whether cortical thickness in occipital and parietal regions predicted global motion processing in 7-year-old children born very preterm. Structural magnetic resonance imaging (MRI) measures were successfully computed for 99 children, motion coherence thresholds were evaluated in 85 participants and functional MRI blood oxygen level-dependent (BOLD) percent signal change in the primary visual cortex (V1) and/or middle temporal area (V5) was quantifiable in 23 participants. Parietal cortical thickness predicted V5 BOLD response to 100% coherent motion stimuli, where individuals with thinner parietal cortices had stronger V5 BOLD responses to coherent motion. These findings indicate that parietal thickness may serve as a structural indicator of dorsal stream development in children born very preterm and provide a structural basis for understanding individual differences in global motion processing.

## Introduction

Very preterm birth (<32 weeks’ gestation) and very low birth weight (<1,500 g) can lead to altered brain development, including changes in cortical structure and function (de Kieviet et al. 2012). Children born very preterm are at greater risk of refractive error, strabismus, and abnormal stereopsis, as well as impairments in cortical visual processing that supports global motion perception and visuomotor integration (Leung et al. 2018) than those born at term. Very preterm infants have considerably higher protein requirements than infants born at term and commonly receive parenteral amino acid solutions to meet these needs during the neonatal period (Hay and Thureen 2010; Embleton and van den Akker 2019). Findings from the Protein, Insulin, And Neonatal Outcomes (PIANO) Study show that in 7-year-old children born very preterm, optimizing protein intake during the neonatal period is a promising strategy for supporting development of the brain and visual system (Kulmaganbetov et al. 2023; Nguyen et al. 2025; Poppe et al. 2025).

Individuals born preterm display delayed cortical thinning during early to late childhood since synaptic pruning, the elimination of unnecessary neural connections, occurs more slowly compared to their full-term peers (Mürner-Lavanchy et al. 2014). Infants born preterm also demonstrate delayed cortical expansion, possibly due to disruptions in the third trimester caused by premature birth that perturb the movement of thalamocortical axons and GABAnergic neurons into the cortex (Kostović and Jovanov-Milošević 2006; Volpe 2009). Consequently, cortical thickness is greater in parietal, temporal, and frontal regions (Bjuland et al. 2013; Mürner-Lavanchy et al. 2014; Nam et al. 2015; Dimitrova et al. 2021), and cortical surface area is generally reduced (Phillips et al. 2011; Skranes et al. 2013; Makropoulos et al. 2016) in preterm individuals across studies spanning infancy to adulthood. Such alterations in cortical structure may influence the development of visual processing (Klaver et al. 2015).

Global motion perception, the ability to integrate local motion signals into an overall direction of motion, is a visual function that is commonly tested in studies of visual development in children born with neurodevelopmental risk factors including prematurity (Atkinson and Braddick 2007; Taylor et al. 2009). This is because pediatric disorders can preferentially impair development of the dorsal visual pathway, which includes brain regions that are specialized for global motion processing (Atkinson 2017) and that support visuo-motor control. The dorsal visual pathway starts in the primary visual cortex (V1), where responses to directional motion occur within local receptive fields (Hubel and Wiesel 1968). It then travels to the motion-sensitive middle temporal visual area (V5). Global motion processing depends critically on V5 because neurons in this area integrate low-level, local representations of luminance changes over time into a coherent, global perception of motion (Movshon and Newsome 1996; Braddick et al. 2003). The dorsal stream terminates in the parietal lobe and provides visual information critical for visual-motor integration (Felleman and Van Essen 1991; Goodale and Milner 1992). Dorsal pathway responses are also modulated by top-down signals from the parietal cortex (Ruff et al. 2008; Silvanto et al. 2009). The posterior parietal cortex is part of a network of brain areas that supports visual attention and provides attentional modulation of V1, as well as the connections from V2 to V5 (Friston and Büchel 2000; Jack et al. 2006).

Global motion perception can be assessed psychophysically using the motion coherence threshold: the minimum proportion of dots moving in a common direction required to correctly identify the overall (global) motion direction (Braddick 1995). Because impaired global motion perception reflects impaired development of the dorsal visual pathway, motion coherence thresholds are commonly used to probe dorsal stream function (Pellicano and Gibson 2008; Chakraborty et al. 2017; Leung et al. 2018). In children, better motion coherence thresholds have been associated with reduced cortical thickness of V1 (Bhat et al. 2021) and with a relative increase in parietal area (Braddick et al. 2016).

Global motion perception can also be assessed neurophysiologically by using functional magnetic resonance imaging (fMRI) to assess blood oxygen level-dependent (BOLD) changes in visual brain areas while participants view global motion stimuli. While V1 is activated equally by incoherent motion (a field of dots moving randomly) and coherent motion (a proportion of dots within a field moving with the same direction and speed), V5 can be more strongly activated by coherent motion (Rees et al. 2000; Braddick et al. 2001; Aspell et al. 2005; Hesselmann et al. 2008), although there is mixed evidence (McKeefry et al. 1997; Lam et al. 2000). These patterns are consistent with the roles of V1 and V5 in local motion signaling and motion integration, respectively.

The PIANO Study followed a well-characterized cohort of 7-year-old children born very preterm, who received parenteral nutrition before or after a protocol change intended to increase early protein intake (Tottman et al. 2020). Previous findings from this cohort have linked enhanced neonatal protein intake to thinner (more mature) lateral occipital and lateral parietal cortices (Poppe et al. 2025), better motion coherence thresholds (Kulmaganbetov et al. 2023), and larger visual area V5 neural responses to coherent motion (Nguyen et al. 2025). However, each of these studies examined structural or functional outcomes independently, and whether cortical thickness in these regions is associated with global motion processing in this population remains unknown. Establishing such a structure–function association could provide an anatomical basis for individual differences in global motion processing in children born very preterm.

This study, therefore, examined whether occipital and parietal cortical thickness predicted global motion processing in the PIANO cohort. We used structural MRI to quantify cortical thickness, psychophysics to measure motion coherence thresholds, and fMRI to measure V5 BOLD responses to coherent motion. Based on the architecture of the dorsal visual pathway and the known involvement of the occipital and parietal cortices in global motion perception, we hypothesized that thinner occipital and parietal cortices would be associated with better behavioral motion coherence thresholds and larger V5 BOLD responses to coherent motion in 7-year-old children born very preterm.

## Materials and methods

### Participants

As previously reported by Tottman et al. (2020) and Duerden et al. (2021), the PIANO Study cohort consists of children born very preterm (24 to 30 weeks’ gestation) who were admitted to the neonatal intensive care unit (NICU) at the National Women’s Hospital, Auckland, New Zealand. The participants were admitted either before (July 2005 to December 2006) or after (January 2007 to October 2008) a change in the neonatal parenteral nutrition protocol. The original parenteral formulation contained amino acids, minerals, and electrolytes, made up in 10% dextrose solution. The new parenteral solution had a higher protein concentration administered with decreased total fluid intake to better meet international recommendations (Tsang 2005; Cormack et al. 2011). Because of the reduced fluid intake, carbohydrate intake was also reduced in the new protocol group (Tottman et al. 2020).

**Figure 1:**
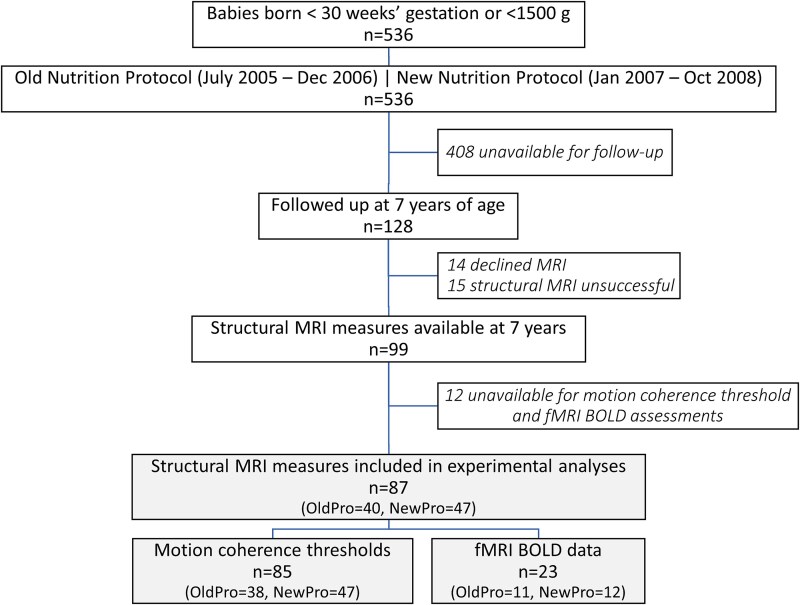
Recruitment of participants for follow-up assessments of structural MRI measures, motion coherence thresholds and fMRI.

Infants who were admitted to the neonatal unit for at least the first postnatal week during the original study were eligible for follow-up at 7 years of age. The exclusion criteria were: did not survive to 7 years’ corrected age, exposed to both old and new nutrition protocols within the first 7 d, or transferred into the NICU after 24 h or transferred out before postnatal day 7. Of the 536 infants who were admitted to either the original or new nutrition protocol (Fig. 1), 128 were followed up at 7 years of age. Of these, 114 consented to MRI and 109 completed at least a subset of the MRI sequences. Structural MRI measures were successfully computed for 99 children, and 87 of these participants also had motion coherences thresholds or fMRI BOLD percent signal change evaluated. Only these 87 children were included in further analyses. Among these children, motion coherence thresholds were evaluated in 85 participants, and fMRI BOLD percent signal change (measured in V1 and/or V5) was quantifiable in 23 participants.

The analyzed sample comprised 42 male and 45 female children. There were 40 children in the OldPro group and 47 in the NewPro group. Further participant characteristics, including group-level differences in nutritional intake, are reported in the Results.

Ethics approval was given by the Northern B ethics committee (NTY/12/05/035) and the Auckland District Health Board (ADHB 5486). Written informed consent was provided by the parents or legal guardians of the participants, and verbal assent was given by the children.

### Stimuli

Global motion perception was assessed using random dot kinematograms (RDKs). RDKs were constructed from a population of moving dots. A percentage of the dots moved together in one coherent, global direction and the rest moved randomly. The RDK parameters were the same for both MRI and psychophysical measurements. RDKs were composed of 100 circular white dots (137 cd/m^2^), and each dot had a diameter of 0.24°. The dots had a limited lifetime, with each dot having a 5% chance of disappearing in each frame and reappearing in a random location. The dots moved at a speed of 6°/s, were shown within a 10° diameter circular aperture and were presented on a mean-luminance gray background (45 cd/m^2^). Each trial lasted 2,000 msec. The RDKs were generated using Matlab (Mathworks) and the Psychophysics Toolbox (Brainard and Vision 1997; Pelli 1997). These stimuli have previously been described by Chakraborty et al. (2015).

### Magnetic resonance imaging

Anatomical and functional MRI were performed on a 3 T scanner (Siemens, Skyra, Erlangen, Germany). Anatomical images were acquired using an MPRAGE pulse sequence ([repetition time] TR, 2,000 ms, [echo time] TE, 3,510 ms, [inversion time] TI, 1,010 ms, slice thickness, 0.85 mm, [field of view] FOV, 210 × 210 mm, matrix, 176 × 256 × 256) (Duerden et al. 2021). Functional images were acquired using BOLD contrast T2^*^-weighted echo planar imaging ([repetition time] TR, 2,500 ms, [echo time] TE, 27 ms, FOV, 147 × 147 mm, matrix, 64 × 64, slice thickness, 3 mm, 48 slices, flip angle of 90 degrees in a phase-encoding direction of anterior to posterior).

The fMRI procedure used a block design which included four conditions: “blank,” “100% coherent,” “0% coherent,” and “static.” The “blank” condition showed a fixation stimulus on a mean luminance screen, the “100% coherent” condition showed RDK stimuli alternating between all dots moving upwards and all dots moving downwards, the “0% coherent” condition showed the RDK dots moving in random directions, and the “static” condition showed stationary limited-lifetime (“twinkling”) RDK dots. “Blank” blocks were shown only at the beginning and end of the scan. Three “100% coherent” blocks and three “0% coherent” blocks were randomly sequenced and interspersed with baseline “static” blocks. Each block lasted 20 s and contained 20 trials. During each trial, the RDK stimulus was presented for 750 ms followed by 250 ms of blank fixation. The stimuli were back-projected onto a screen behind the scanner bore and participants viewed the stimuli through a slanted mirror placed above the head coil, at a distance of 60 cm. Each participant completed a single run of the paradigm, comprising 13 blocks of 20 s each, for a total run duration of 260 s and 104 functional volumes acquired.

The fMRI data were preprocessed using BrainVoyager 22.4 software and its embedded Python interpreter. Preprocessing of functional data included slice timing correction, motion correction, and temporal high-pass filtering. EPI distortion correction was not applied because field maps were difficult to acquire in the child participants. Preprocessing of anatomical data included iso-voxel transformation, spatial intensity inhomogeneity correction, and brain extraction. Co-registration of functional and anatomical data was checked manually and corrected where necessary. Data were transformed to MNI (Montreal Neurological Institute) space.

**Figure 2:**
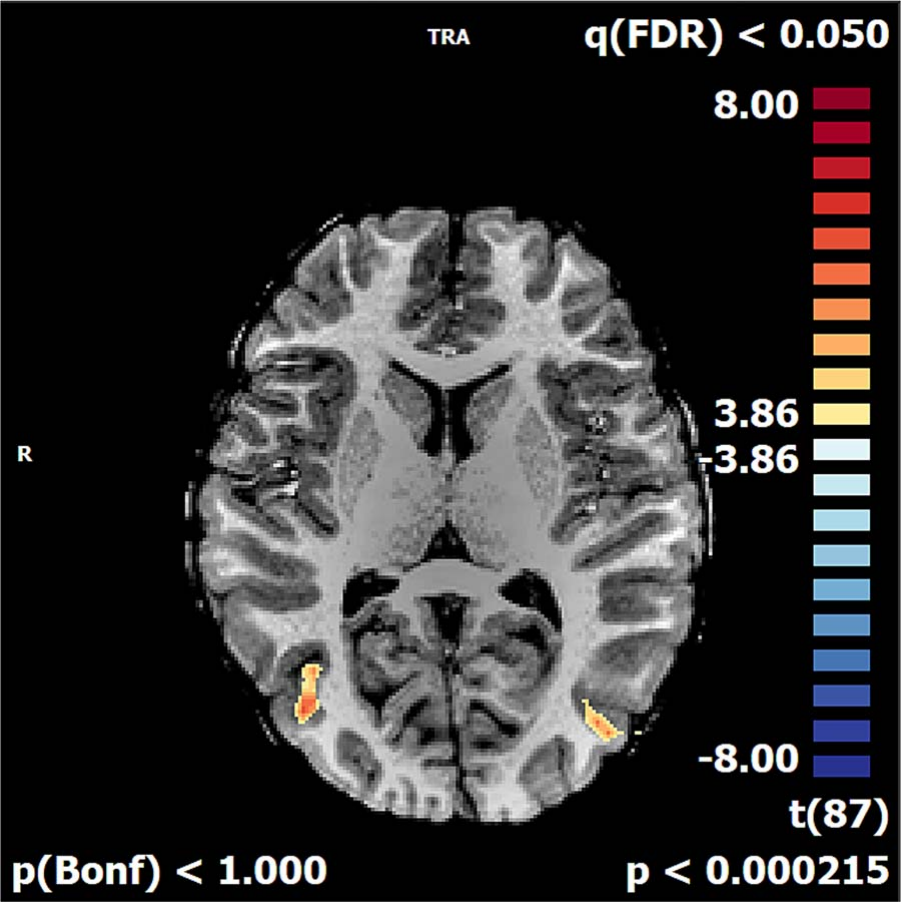
Example of V5 activation from a study participant. Right and left V5 were identified as clusters of voxels that showed significantly greater BOLD activity to moving stimuli (0% and 100% coherent RDK) versus static stimuli. Activation maps for all participants are provided in Supplementary Fig. S1.

A general linear model (GLM), containing z-scored head motion nuisance regressors, was fit to identify brain voxels that showed significantly greater BOLD activity to moving stimuli (0% and 100% coherent) versus static stimuli. Areas of activation were corrected for multiple comparisons using the false discovery rate (q < 0.05). For each hemisphere, the V1 region of interest (ROI) was defined as a significant cluster of voxels located within the V1 area defined by the Juelich Histological atlas (Wilms et al. 2005; Malikovic et al. 2007). Also in each hemisphere, the V5 ROI was defined as a significant cluster of voxels located near the ascending limb, or the posterior continuation, of the inferior temporal sulcus or the posterior bank of the superior temporal sulcus (Fig. 2) (Dumoulin et al. 2000).

The BOLD percent signal change within each ROI, for both the “0% coherent” and “100% coherent” conditions, was calculated using event-related averaging. Each motion block was preceded by a “static” block, which served as a baseline. To calculate the average baseline for each scanning run, the 7.5 s immediately preceding each motion block was averaged across all motion blocks to give a baseline. The BOLD percent signal change was then calculated as: (*value—baseline*)*/baseline.* Percent signal change values within a time window of 20 s, starting 5 s after stimulus onset, were averaged to calculate the percent signal change for each condition and for each ROI. For each participant, the BOLD percent signal changes for the right and left hemisphere ROIs were then averaged for each ROI under each stimulus condition. If an ROI for V1 or V5 could only be identified in one hemisphere, only data from that hemisphere were analyzed for that visual area.

The classification of tissue types, structures, and cortical labeling was performed using the recon-all pipeline of the FreeSurfer 6.0 image analysis suite (http://surfer.nmr.mgh.harvard.edu/) (Fischl, 2012). Data were inspected and manually corrected if necessary and resubmitted to the recon-all pipeline.

### Motion coherence thresholds

Global motion coherence thresholds were measured in a separate behavioral session outside the MRI scanner using RDKs, as previously reported by Chakraborty et al. (2015). Participants were situated 40 cm from a video graphics array (VGA) convex computer monitor (HP 7540, resolution of 1,024 × 768), and the RDKs were presented within a black square outline, with a fixation dot in the center of the square aperture. The RDKs consisted of two populations of stimulus dots, one which moved up or down coherently, and the second which moved randomly. A 2-down-1-up staircase method was used to estimate motion coherence thresholds. Contrast was fixed at 100%. RDKs began at 100% coherence, and the staircase varied the coherence level. The first proportional step size was 0.5, and the following steps were fixed at a proportion of 0.25. The motion coherence threshold was calculated by averaging the last 4 of 5 reversals.

### Statistical analysis

All statistical analyses were carried out using Python 3.8.18 (with scipy 1.10.1, statsmodels 0.14.0 and pingouin 0.5.3), JASP 0.18.3 and SPSS 29.0.2.0. The Shapiro–Wilk test and Q-Q plots were used to assess if data were approximately normally distributed. Levene’s test was used to test for homogeneity of variances.

To compare nutritional intake between the OldPro and NewPro groups, *P*-values were calculated based on the assumptions of normality and homogeneity of variances. If the normality and homogeneity assumptions were both met, an independent *t-*test was conducted to compare the mean values between groups. If the normality assumption was met but the homogeneity assumption was not, Welch’s *t-*test was used. If neither the normality nor the homogeneity assumptions were met, the Mann–Whitney U test was used for the comparison.

Note that the analyses described below involved slightly different groups because some children only had data available for two of the three outcome measures (cortical structure, V1 and/or V5 functional responses to global motion stimuli and motion coherence thresholds; Fig. 1).

To compare with previous studies of this cohort in which regional cortical areas, regional cortical thicknesses, and motion coherence thresholds were assessed across all participants (Kulmaganbetov et al. 2023; Poppe et al. 2025), we also evaluated these metrics in the subset of participants that met the criteria for inclusion in the current analysis. Analysis of covariance (ANCOVA) models were used to compare each metric between the OldPro and NewPro groups. Socioeconomic status (New Zealand Deprivation Index, NZDep) (Atkinson et al. 2014) was included as a covariate since it differed by >10% between groups (see Supplementary Table S1). Group (OldPro/NewPro) was also included as a covariate to control for the noncontemporaneous nature of the cohorts.

Multiple linear regression models were run with data from the OldPro and NewPro groups combined to test for structure–function associations. For fMRI data, BOLD percent signal change was modeled using four predictors: occipital area and parietal area (adjusted for overall cortical area), and parietal thickness and occipital thickness (adjusted for mean cortical thickness). Sex (female/male), birthweight z-score, and socioeconomic status were included as covariates, since they differed by >10% between groups (see Supplementary Table S2). Group was also included as a covariate. To satisfy the assumption of normally distributed residuals, BOLD percent signal change values were log-transformed. For each predictor, separate analyses were conducted for BOLD percent signal change in V1 and V5, each at 0% and 100% RDK coherence.

For behavioral data, motion coherence thresholds were also modeled using the same four predictors: occipital area and parietal area (adjusted for cortical area), and parietal thickness and occipital thickness (adjusted for mean cortical thickness). Socioeconomic status was included as a covariate since it differed by >10% between groups (see Supplementary Table S1) and group was also included. To satisfy the assumption of normally distributed residuals, motion coherence threshold values were log-transformed.

Each multiple linear regression model was executed again, with an interaction term between group and the predictor to examine whether the predictor’s association with the outcome differed between the OldPro and NewPro groups. If an interaction effect between group and the predictor was found for any of the regression models, the original model was rerun separately for each group, with group removed as a covariate.

If any dependent variable showed a significant association in the above multiple linear regression models, a new model was constructed that maintained the same dependent variable and covariates, while incorporating the temporal lobe (ventral stream) area or thickness as the predictor variable. This was to assess whether significant associations were specific to the dorsal stream occipital and parietal regions, or if there was a more generalized relationship across different cortical regions that extended to areas associated with the ventral processing stream.

Given the observed group differences in lateral occipital and lateral parietal thickness (Poppe et al. 2025), any dependent variable that showed a significant association with overall occipital or parietal thickness had a new model constructed with lateral occipital or lateral parietal thickness as the predictor. This was to evaluate whether the subregions of lateral occipital and lateral parietal thickness uniquely influenced the dependent variable, distinct from the broader regions of overall occipital and parietal thickness. The new model included the original covariates and was run with and without a group interaction term.

To account for the risk of Type 1 errors due to multiple comparisons, the significance levels were adjusted using the Benjamini–Hochberg procedure, with the false discovery rate (FDR) level set at 0.05. To address limitations in statistical power in the regression models involving BOLD responses, arising from the inclusion of multiple covariates and a limited sample size, sensitivity analyses were conducted with sex, birth weight, and socioeconomic status excluded from these models.

## Results

### Participant characteristics

Infants in the NewPro group had a higher protein-to-energy ratio in days 0 to 7 and days 0 to 14, higher percentage of total protein received parenterally in days 0 to 7 and days 0 to 14, higher intake of protein in the first week after birth, lower intake of carbohydrate in the first week and first month after birth, and lower energy intake in the first month after birth than participants in the OldPro group (Table 1).

**Table 1:** Neonatal nutritional intake of children exposed to the original (OldPro) and new (NewPro) neonatal parenteral nutrition protocols.

	OldPro group (*n* = 40)	NewPro group (*n* = 47)	*P*-value: OldPro vs. NewPro
**P:E ratio (g/kcal): Days 0 to 7**	2.78 ± 0.26	3.62 ± 0.41	<0.001^*^
**P:E ratio (g/kcal): Days 0 to 14**	2.71 ± 0.13	3.14 ± 0.32	<0.001^*^
**% Total protein received parenterally: Days 0 to 7**	0.72 ± 0.20	0.82 ± 0.14	0.013^*^
**% Total protein received parenterally: Days 0 to 14**	0.39 ± 0.21	0.53 ± 0.21	0.002^*^
**Protein: Week 1 (g/kg/day)**	2.38 ± 0.33	2.95 ± 0.37	<0.001^*^
**Protein: Month 1 (g/kg/day)**	3.37 ± 0.26	3.45 ± 0.25	0.138
**Carbohydrate: Week 1 (g/kg/day)**	11.81 ± 1.53	10.28 ± 1.46	<0.001^*^
**Carbohydrate: Month 1 (g/kg/day)**	15.45 ± 1.22	14.53 ± 1.18	<0.001^*^
**Fat: Week 1 (g/kg/day)**	3.66 ± 0.82	3.59 ± 0.78	0.672
**Fat: Month 1 (g/kg/day)**	6.27 ± 0.54	5.92 ± 0.84	0.101
**Energy: Week 1 (kcal/kg/day)**	85.56 ± 10.61	81.92 ± 9.87	0.082
**Energy: Month 1 (kcal/kg/day)**	130.19 ± 10.00	123.59 ± 12.99	0.022^*^

Data are presented as mean ± standard deviation. P:E ratio = protein-to-energy ratio. Asterisks (*) denote significant differences between groups (*p* < 0.05).

**Figure 3:**
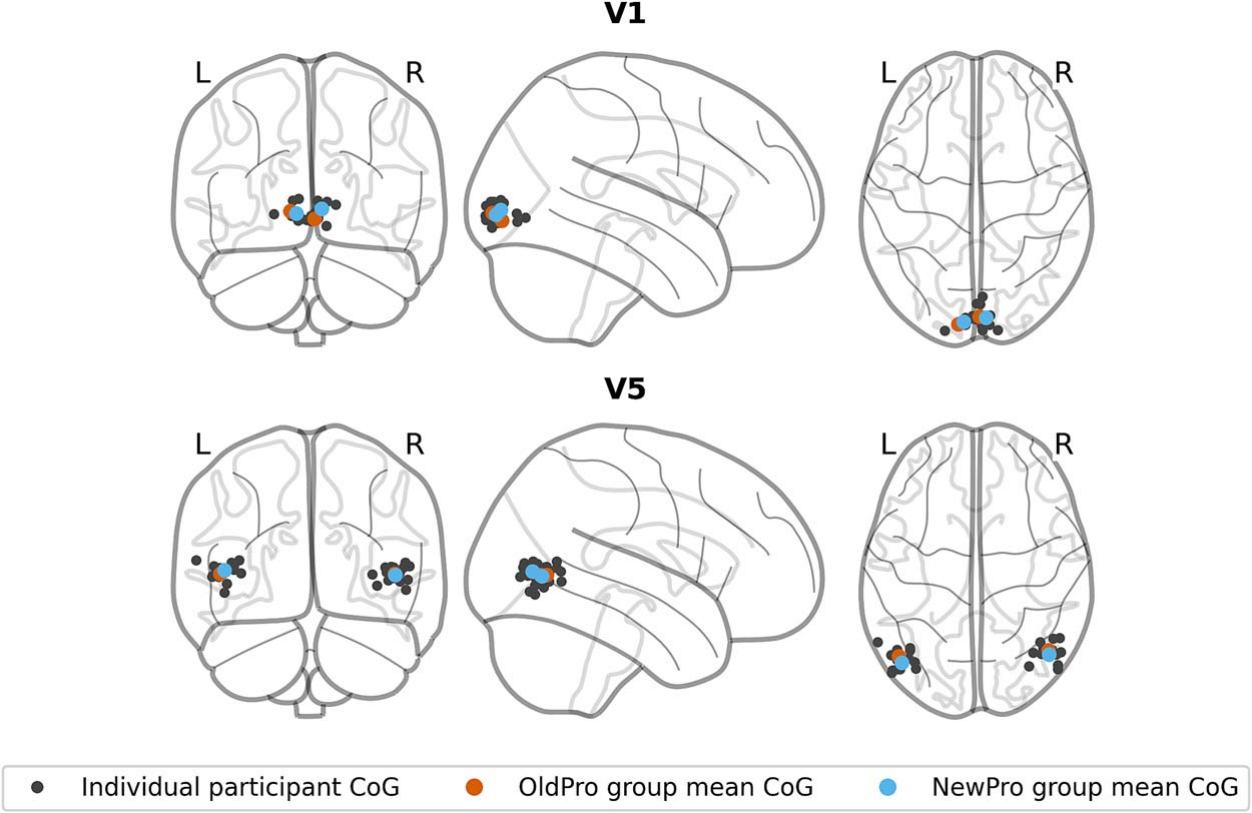
CoG locations for V1 and V5 fMRI ROIs for the 23 children in whom fMRI BOLD percent signal change was quantifiable, displayed on a standard MNI glass brain. Markers indicate individual participant CoGs and the OldPro and NewPro group mean CoGs, as defined in the figure legend. CoGs were derived from voxels showing significantly greater BOLD activity to moving versus static stimuli (FDR q < 0.05). Exact coordinates and standard deviations are reported in Table S3 in the supplementary.

V1 and V5 ROIs were defined per participant using a GLM. Not all participants had ROIs identified in both hemispheres (see Supplementary Fig. S1). Figure 3 shows the centers of gravity (CoG) of ROIs for individual participants and group means. V1 CoGs were located in the posterior occipital cortex and V5 CoGs were located in the lateral occipitotemporal cortex, consistent with the known locations of these visual areas.

**Figure 4:**
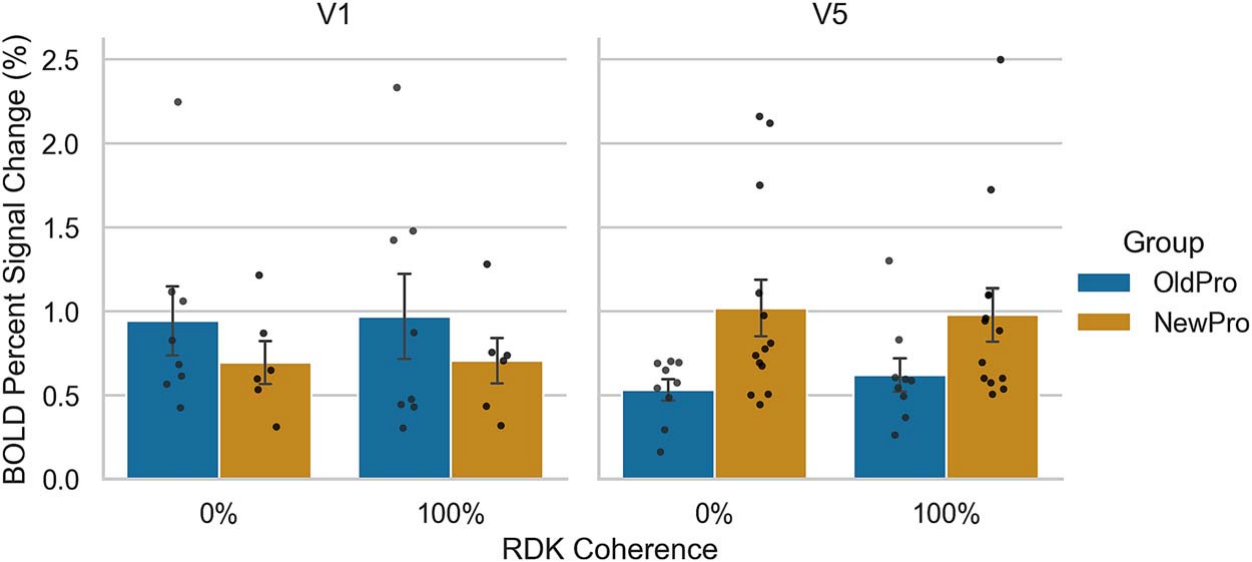
BOLD percent signal change for V1 and V5 in response to 0% and 100% coherent motion in the OldPro and NewPro groups. Bars represent the group mean and error bars represent the standard error of the mean. Individual data points represent the BOLD percent signal change for each participant.

BOLD percent signal change within each ROI was calculated using event-related averaging and is shown for individual participants and group means in Fig. 4. Both V1 and V5 showed positive responses to 0% and 100% coherent motion in OldPro and NewPro participants.

### Group differences in brain structure and motion processing

Regional cortical areas, regional cortical thicknesses, and motion coherence thresholds were assessed in our subset of participants, in order to compare with previous studies of this cohort that assessed these variables in isolation and therefore included larger subsets of the available cohort (Kulmaganbetov et al. 2023; Poppe et al. 2025). In agreement with these previous studies, our subset exhibited significantly thinner lateral occipital and lateral parietal cortices in the NewPro than OldPro group (Table S4, Fig. S2). Motion coherence thresholds were significantly lower (better) in the NewPro than OldPro group (Fig. S3).

### Associations between cortical thickness and blood oxygen level-dependent responses

**Figure 5:**
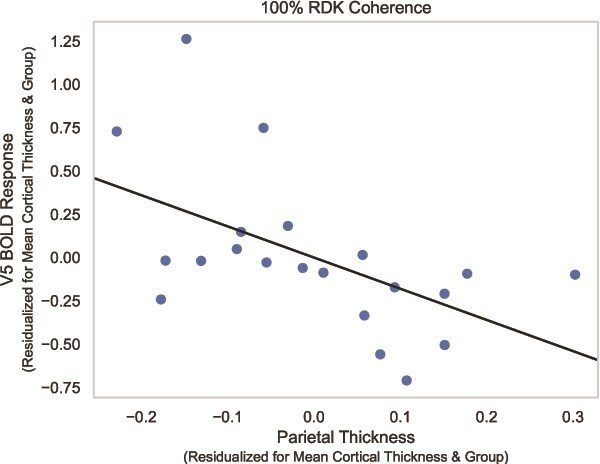
Partial regression plot illustrating the relationship between V5 BOLD percent signal change and parietal thickness at 100% RDK coherence, while controlling for mean cortical thickness and group. Thinner cortex was associated with a larger BOLD response. The plot displays residuals with a linear regression line, demonstrating the independent association between V5 BOLD percent signal change and parietal thickness after accounting for covariates. Note that the adjusted V5 BOLD response is based on raw data. For statistical analysis, the V5 BOLD percent signal change was log-transformed to satisfy model assumptions (refer to Fig. S4 in the supplementary).

Multiple linear regression models were used to examine the relationship between log-transformed BOLD percent signal change and either occipital or parietal thickness. These were assessed for V1 and V5 BOLD responses to incoherent and coherent motion. In the fully adjusted models with all covariates included (Table 2), occipital thickness was negatively associated with V1 BOLD response at 0% and 100% RDK coherences, and with V5 BOLD response at 100% RDK coherence. Parietal thickness was negatively associated with V1 BOLD response at 100% RDK coherence, and with V5 BOLD response at 100% RDK coherence. In the sensitivity analyses using simplified models, which included only mean cortical thickness and group as covariates (sex, birth weight, and socioeconomic status were removed as covariates), only the association between parietal thickness and V5 BOLD response at 100% RDK coherence remained significant (Table 3, Fig. 5).

**Table 2:** Full multiple linear regression analyses predicting BOLD percent signal change (log-transformed) as a function of occipital thickness or parietal thickness.

Predictor variable	Brain area	RDK coherence	β (95% CI)	t	p	Corrected p (FDR)	R-squared	Adjusted R-squared
**Occipital thickness**	**V1**	**0%**	−1.594 (−2.450, −0.738)	−4.403	0.003^*^	0.013^*^	0.790	0.609
		**100%**	−1.775 (−2.484, −1.066)	−5.919	0.001^*^	0.005^*^	0.856	0.732
	**V5**	**0%**	−0.349 (−1.302, 0.604)	−0.785	0.446	0.491	0.330	0.043
		**100%**	−1.109 (−1.904, −0.314)	−2.993	0.010^*^	0.017^*^	0.534	0.335
**Parietal thickness**	**V1**	**0%**	−1.025 (−2.514, 0.464)	−1.627	0.148	0.197	0.424	−0.069
		**100%**	−1.622 (−2.735, −0.508)	−3.444	0.011^*^	0.017^*^	0.678	0.402
	**V5**	**0%**	−0.407 (−1.644, 0.829)	−0.707	0.491	0.491	0.324	0.035
		**100%**	−1.415 (−2.449, −0.380)	−2.932	0.011^*^	0.017^*^	0.527	0.324

BOLD percent signal change was measured in V1 and V5, in response to RDK coherences of 0% and 100%. Associations were adjusted for mean cortical thickness, birth weight (z-scored), sex, socioeconomic status, and group. Standardized coefficients (β), standardized confidence intervals, t-values, *P*-values, corrected *P*-values, r-squared values, and adjusted r-squared values are reported. Bold text indicates the grouping categories: predictor variable, brain area, and RDK coherence level. Asterisks (^*^) indicate significant associations (*P* < 0.05).

**Table 3:** Simplified multiple linear regression analyses (sensitivity analyses) predicting BOLD percent signal change (log-transformed) as a function of occipital thickness or parietal thickness.

Predictor variable	Brain area	RDK coherence	β (95% CI)	t	p	Corrected p (FDR)	R-squared	Adjusted R-squared
**Occipital thickness**	**V1**	**0%**	−0.732 (−1.501, 0.037)	−2.120	0.060	0.120	0.417	0.242
		**100%**	−0.690 (−1.538, 0.159)	−1.811	0.100	0.160	0.290	0.077
	**V5**	**0%**	−0.116 (−0.849, 0.618)	−0.333	0.743	0.743	0.281	0.154
		**100%**	−0.668 (−1.359, 0.023)	−2.038	0.057	0.120	0.361	0.248
**Parietal thickness**	**V1**	**0%**	−0.851 (−2.033, 0.331)	−1.604	0.140	0.186	0.327	0.126
		**100%**	−1.316 (−2.365, −0.267)	−2.796	0.019	0.076	0.471	0.312
	**V5**	**0%**	−0.433 (−1.489, 0.623)	−0.865	0.399	0.456	0.307	0.185
		**100%**	−1.352 (−2.247, −0.457)	−3.187	0.005	0.043^*^	0.502	0.414

BOLD percent signal change was measured in V1 and V5 in response to RDK coherences of 0% and 100%. Associations were adjusted for mean cortical thickness and group. Standardized coefficients (β), standardized confidence intervals, t-values, *P*-values, corrected *P*-values, r-squared values, and adjusted r-squared values are reported. Bold text indicates the grouping categories: predictor variable, brain area, and RDK coherence level. Asterisks (^*^) indicate significant associations (*P* < 0.05).

### Additional analyses

Multiple linear regression models were used to examine the relationship between log-transformed BOLD percent signal change and either occipital or parietal area (Table S5). Regression models were also used to examine the relationship between log-transformed motion coherence thresholds (measured psychophysically) and four brain structure measurements: occipital area, parietal area, occipital thickness, and parietal thickness (Table S6). No significant associations were observed for any of these models.

For each of the previous multiple linear regression models, an interaction term between group and the predictor was added to assess whether the relationship between the predictor and outcome differed between the OldPro and NewPro groups. No significant group interaction effects were evident (Tables S7 to S9).

To test whether the significant associations found with BOLD response were specific to occipital and parietal thickness, or if there was a more generalized relationship across different cortical regions, regression analyses were performed with temporal lobe thickness predicting BOLD response (Table S10). Also, to assess whether the subregions of lateral occipital and lateral parietal thickness uniquely influenced BOLD response, regression analyses were performed with lateral occipital and lateral parietal thickness predicting BOLD response, with and without a group interaction term (Tables S11 and S12). No significant associations were found for any of these multiple linear regression models.

## Discussion

### Study context and confirmation of group differences

The aim of this study was to explore potential associations between cortical structure and functional measures of global motion processing in 7-year-old children born very preterm. Earlier studies have assessed cortical structure and global motion processing separately in subsets of the same study cohort, examining group differences across participants. Poppe et al. (2025) examined cortical structure and observed thinner lateral occipital and lateral parietal cortices in the NewPro group. For global motion processing, Nguyen et al. (2025) found a larger V5 BOLD response to coherent motion and Kulmaganbetov et al. (2023) found better motion coherence thresholds in the NewPro group. We carried out between-group comparisons for cortical structure and global motion processing in our subset of participants from the same PIANO cohort, and the results were consistent with those of the above studies. The alignment of our findings with those from past studies reinforces the idea that early nutrition influences brain maturation and dorsal stream development in children born very preterm.

While the previous studies linked neonatal protein intake to structural or functional outcomes independently, none examined whether cortical structure and visual function are associated. The present study addressed this gap by assessing whether variability in cortical structure predicted global motion processing. Establishing such a structure–function relationship would provide a structural basis for understanding individual differences in global motion processing. This could offer new insight into how cortical maturation shapes dorsal stream function in children born very preterm.

### V5 activity and parietal cortical thickness

Parietal thickness predicted V5 BOLD response magnitude during presentation of 100% coherent motion stimuli. This may be attributable to the reciprocal connections between V5 and the parietal cortex in the dorsal visual pathway, where the parietal lobe serves as a major convergence point. When V5 responds to global motion, it projects signals forward to the parietal cortex. In turn, the parietal cortex sends feedback signals to modulate the strength of effective connectivity between early visual cortex and V5 (Friston and Büchel 2000; Bressler et al. 2008). This modulation is (i) dependent on current visual input and occurs only in the presence of moving visual stimuli (Ruff et al. 2008; Ruff et al. 2009) and (ii) regulated by attention, translating to increased connectivity when visual attention is engaged (Friston and Büchel 2000). Since the parietal lobe receives sensory signals from V5 when global motion processing occurs and sends feedback signals to modulate this processing when attention is engaged, a more mature (thinner) parietal cortex structure may support motion processing in V5.

Parietal thickness predicted V5 BOLD response specifically at 100% motion coherence, which may reflect optimized attentional modulation at this coherence level. Modulatory effects from the parietal cortex are driven by attention, and a 100% coherent RDK provides a unified moving pattern that strongly captures and holds visual attention compared to random motion. Better sustained attention at this coherence level may lead to stronger modulatory influences from the posterior parietal cortex, leading to a greater association between parietal structure and V5 BOLD response.

The association between parietal thickness and V5 BOLD response was negative, indicating that V5 BOLD response increases as the parietal cortex becomes thinner. This is consistent with previous studies which have demonstrated that a thinner cortex is linked with better performance. Bhat et al. (2021) showed that motion coherence sensitivity increased with decreasing cortical thickness of V1. Also, examination of the full PIANO cohort by Poppe et al. (2025) found that participants with thinner lateral occipital and lateral parietal cortices had a more mature profile of brain metrics. Thinner cortices may reflect improved brain maturation, considering that cortical thickness reduction occurs during childhood via synaptic pruning of inefficient connections (Knudsen 2004). This process appears to occur more slowly in children born preterm than in controls, as cortical thinning continues between the ages of 7 and 12 years in children born preterm but appears to be mostly completed before the age of 7 years in controls (Mürner-Lavanchy et al. 2014). Thus, our 7-year-old participants with thinner parietal cortices may have experienced less disruption to cortical thinning, resulting in more refined neural circuits and a stronger V5 BOLD response.

There was no group interaction effect, indicating that the association between parietal thickness and V5 BOLD response was consistent across the OldPro and NewPro groups. As previously mentioned, participants in the NewPro group had thinner lateral parietal cortices and larger V5 BOLD responses. These patterns suggest that neonatal protein intake may shift structural and functional development towards improved outcomes, while preserving the fundamental relationship between parietal structure and V5 activity. Since global motion perception is commonly used to assess dorsal stream function, its stable association with parietal thickness across treatment groups indicates that parietal thickness can potentially be used as a structural indicator of dorsal stream development, regardless of neonatal intervention history.

### Additional structural-functional relationships in visual areas

We found other associations in the fully adjusted linear regression models that did not remain significant in the sensitivity analyses using simplified models. Occipital thickness predicted V1 BOLD response during presentations of both 0% and 100% coherent RDKs. V1 is located within the occipital cortex; therefore, these results indicate that local cortical structure is associated with functional responses. Furthermore, V1 is less specialized than regions further along the dorsal processing stream, and it contains neurons with small, local receptive fields that respond to directional motion (Hubel and Wiesel 1968; Braddick et al. 2003). As such, V1 responds to directional motion signals present in both incoherent and coherent stimuli, which may explain why associations were found at both 0% and 100% motion coherence.

Occipital thickness also predicted V5 BOLD response during presentation of 100% coherent motion stimuli only. This association is consistent with the role of V5 in coherent motion processing, in which signals from early visual areas in the occipital lobe are integrated into a global perception of motion (Riečanskỳ 2004). V5 tuning for coherent motion detection is supported by studies which have reported greater V5 activation by coherent motion compared to noise (Rees et al. 2000; Braddick et al. 2001; Aspell et al. 2005).

Parietal thickness predicted V1 BOLD response during presentation of 100% coherent RDKs. V1 is modulated by attention and receives feedback from the parietal cortex when attention is engaged (Friston and Büchel 2000; Jack et al. 2006). As previously mentioned, coherent motion may be better at sustaining attention because the signal is clear and unified. In contrast, incoherent motion does not provide a salient pattern to anchor attention. Therefore, the association between parietal thickness and V1 BOLD response may correspond to modulation by the parietal cortex, which occurs when spatial attention is engaged at 100% RDK coherence.

### Absence of behavioral associations

No associations were found between cortical structure and behavioral motion coherence thresholds. This suggests that parietal structure predicts neural but not behavioral measures of global motion perception. The V5 BOLD response provides a more proximal measure of global motion processing, since it captures neural activity and does not depend on participant feedback. In contrast, the motion coherence threshold requires a behavioral response and may be influenced by factors such as task comprehension, decision-making, and reaction time. Therefore, compared to the V5 BOLD response, the motion coherence threshold may be less sensitive to differences in parietal thickness because it represents a less direct measure of global motion processing.

Earlier findings by Braddick et al. (2016) showed that a relative increase in parietal area and decrease in occipital area were associated with better global motion thresholds (the authors concluded that variation in motion coherence thresholds was linked to the parietal area). A possible reason why we did not replicate these results is that our study focused on 7-year-old children born very preterm, whereas Braddick et al. (2016) examined 5- to 12-year-old typically developing children. The motion coherence thresholds from our participants may have been susceptible to behavioral noise, as discussed above, which may have obscured any association with parietal area. Since the children in Braddick et al. (2016)’s study were healthy and a proportion of them were older than our participants, they may have been able to elicit more mature and stable behavioral responses. Thus, their motion coherence thresholds may have provided a more precise representation of underlying global motion perception, allowing the association between parietal area and motion coherence thresholds to be more easily detectable.

### Limitations and statistical considerations

There are limitations in this study. Our fMRI sample size was relatively small because the long scanning protocol was challenging for 7-year-old children. A considerable number of participants were unable to complete functional scanning, due to discomfort in the MRI environment or falling asleep during the scanning process. Others displayed excessive movement, and ROIs could not be identified in a significant number of children, likely because they were unable to remain engaged with the visual stimulus. These substantial dropout rates may have introduced a bias towards the less affected children. However, the consistent between-group differences in motion coherence thresholds and cortical structure, found in our subset of participants and in previous full cohort analyses (Kulmaganbetov et al. 2023; Poppe et al. 2025), increases our confidence in the findings.

Our two participant groups differed by nutritional protocol (OldPro versus NewPro) but all participants were born very preterm. Consequently, the observed structure–function association may reflect differences in early protein intake but the contribution of prematurity to this relationship could not be directly examined. Therefore, findings from this study should be interpreted within the context of neonatal nutrition, and generalizability beyond the very preterm population remains unknown.

Our small fMRI sample size (*n* = 23) constrained the choice of modeling framework. We considered a linear mixed-effects approach, which would have combined the V1/V5 and 0%/100% coherence measurements in a single model while accounting for within-subject variability. We did not adopt it because a mixed-effects model would have been underpowered at our small sample size. The BOLD analyses were instead conducted as separate regressions per ROI and coherence level, which resulted in a relatively large number of models. To address multiple-testing inflation, we applied Benjamini–Hochberg FDR correction across the primary models.

Our small fMRI sample size also raised the risk of overfitting when all covariates were included in the regression models. We incorporated simplified regression models as sensitivity analyses to address this concern. Most significant associations found in the full regression models did not persist in the simplified models, which may represent statistical overfitting or true weak effects. These associations are considered preliminary and have been interpreted with caution. Replication in larger samples would be needed to provide more definitive conclusions. However, the instability of these associations reinforces the strength of the main V5-parietal thickness association, which was robust enough to remain significant in both the full and simplified regression models.

## Conclusion

In summary, we found that parietal cortical thickness predicted V5 BOLD response at 100% motion coherence, where individuals with thinner parietal cortices had stronger V5 BOLD responses to coherent motion. The association was consistent across the OldPro and NewPro groups, indicating a fundamental relationship between parietal thickness and V5 activity. Since participants in the NewPro group had thinner lateral parietal cortices and larger V5 BOLD responses than those in the OldPro group, this suggests that neonatal protein intake may alter these measures while maintaining the core structure–function relationship. Taken together, the association between parietal cortical thickness and V5 BOLD response, and its consistency across nutritional groups, indicate that parietal thickness may serve as a structural indicator of dorsal stream development in 7-year-old children born very preterm, offering neuroanatomical insight into individual differences in global motion processing in this population. Whether this structure–function relationship extends to typical development remains an important question for future research.

## Supplementary Material

Supplementary_material_bhag089
