# Supplementary material for: Parietal thickness predicts middle temporal area (V5) motion responses in 7-year-old children born very preterm
Source: Cereb Cortex. 2026 Jun 24;36(6):bhag089. doi: 10.1093/cercor/bhag089 (PMC13293252; doi:10.1093/cercor/bhag089)
Supplement: Supplementary_material_bhag089 [file supplementary_material_bhag089.zip › Supplementary.docx]

**Supplementary Materials**

# **Parietal thickness predicts V5 motion responses in 7-year-old children born very preterm**

Linda Nguyen^1^, Andrew E. Silva^1^, Tanya Poppe^2^, Myra Leung^2,3^, Jane M. Alsweiler^4,5^, Joanna Black^2^, Jane E. Harding^6^, Anna C. Tottman^6,7^, Benjamin Thompson^1,2,6,8^, on behalf of the PIANO Study Group

^1^School of Optometry and Vision Science, University of Waterloo, Waterloo, Canada ^2^School of Optometry and Vision Science, University of Auckland, Auckland, New Zealand ^3^Discipline of Optometry and Vision Science, University of Canberra, Canberra, Australian Capital Territory, Australia ^4^Department of Pediatrics: Child and Youth Health, University of Auckland, Auckland, New Zealand ^5^Newborn Services, National Women’s Health, Auckland City Hospital, Auckland, New Zealand ^6^Liggins Institute, University of Auckland, Auckland, New Zealand ^7^Neonatal Services, Royal Women’s Hospital, Melbourne, Australia
^8^Centre for Eye and Vision Research, 17W Science Park, Hong Kong

Corresponding author:
Linda Nguyen
School of Optometry and Vision Science
University of Waterloo
200 University Avenue West
Waterloo, Ontario, N2L 3G1 [linda​.linh​.nguyen@uwaterloo​.ca](mailto:linda.linh.nguyen@uwaterloo.ca)

**Figure S1.** V1 and V5 regions of interest (orange) for each of the 23 participants, overlaid on individual MNI-normalised anatomical scans (axial view). ROIs were defined as significant clusters of voxels showing greater BOLD activity to moving versus static stimuli (FDR q<0.05). n/a indicates the ROI was not identified at this threshold. Participant labels are coloured by nutritional intervention group (blue = OldPro, red = NewPro).

**Figure S2.** Lateral occipital (A) and lateral parietal (B) cortical thickness in the OldPro (n=38) and NewPro (n=47) groups. Bars represent the adjusted group mean and error bars represent the standard error of the mean, both controlling for socioeconomic status. Individual data points represent each participant's raw measurement. Both regions were significantly thinner in the NewPro group than the OldPro group (FDR-corrected p < 0.005), consistent with previous findings in this cohort (Poppe et al. 2025). Full statistical comparisons for all regional cortical area and thickness measures are reported in Table S4.

**Figure S3.** The adjusted means of motion coherence thresholds for the OldPro (n=38) and NewPro (n=47) groups. The means have been adjusted for socioeconomic status. Error bars represent the standard error of the adjusted means. Individual data points represent motion coherence thresholds for each participant. The adjusted mean motion coherence thresholds were OldPro = 57.27 ± 3.84 and NewPro = 45.06 ± 3.44. The difference between groups was significant (F(1, 80) = 5.500, p = 0.021, *η*2 = 0.063).

**Figure S4**: Partial regression plot illustrating the relationship between V5 BOLD percent signal change and parietal thickness at 100% RDK coherence, while controlling for mean cortical thickness and group. The plot displays residuals with a linear regression line, demonstrating the independent association between V5 BOLD percent signal change (log-transformed) and parietal thickness after accounting for covariates. The adjusted V5 BOLD response is based on data that was log-transformed to satisfy model assumptions. This figure complements Figure 5 in the Results section, where the adjusted V5 BOLD response is based on raw data.

**Table S1:** Characteristics of participants who had measurements for both 1) motion coherence thresholds and 2) MRI structural measurements.

| **Characteristic** |  | **OldPro Group (n=38)** | **NewPro Group (n=47)** | **OldPro vs NewPro p-value** |
| --- | --- | --- | --- | --- |
| **Sex (Male)** |  | 18 (47.37%) | 24 (51.06%) | 0.735 |
| **Birth Weight (z-scored)** |  | 0.17 (-0.63, 0.49) | 0.40 (-0.58, 0.71) | 0.179 |
| **Socioeconomic Status** | **Least Deprived** | 9 (23.68%) | 1 (2.13%) | 0.084 |
|  | **Most Deprived** | 3 (7.89%) | 7 (14.89%) | 0.084 |

Only socioeconomic status differed by more than 10% between groups, so was included as a covariate in linear regression analyses. Data are n (%) and median (interquartile range). The chi-square test was used to evaluate differences in proportions and the Mann-Whitney U test was used to assess the difference between median values.

**Table S2:** Characteristics of participants who had measurements for both 1) fMRI BOLD responses and 2) MRI structural measurements.

| **Characteristic** |  | **OldPro Group (n=11)** | **NewPro Group (n=12)** | **OldPro vs NewPro p-value** |
| --- | --- | --- | --- | --- |
| **Sex (Male)** |  | 4 (36.36%) | 3 (25.00%) | 0.554 |
| **Birth Weight (z-scored)** |  | -0.27 (-0.64, 0.21) | 0.30 (-0.12, 0.67) | 0.103 |
| **Socioeconomic Status** | **Least Deprived** | 2 (18.18%) | 0 (0.00%) | 0.449 |
|  | **Most Deprived** | 1 (9.09%) | 1 (8.33%) | 0.449 |

These characteristics were included as covariates in linear regression analyses because they all differed by more than 10% between groups. Data are n (%) and median (interquartile range). The chi-square test was used to evaluate differences in proportions, and the Mann-Whitney U test was used to assess the difference between median values.

**Table S3.** Mean centre of gravity for V1 and V5 fMRI ROIs for the 23 children in whom fMRI BOLD percent signal change was quantifiable.

| **Group** | **ROI** | **Hemisphere** | **Mean (SD) x** | **Mean (SD) y** | **Mean (SD) z** | **Mean (SD) voxel number** | **Participants (n)** |
| --- | --- | --- | --- | --- | --- | --- | --- |
| **OldPro** | **V1** | **Left** | -10.20 (7.51) | -93.67 (3.98) | -0.58 (6.00) | 162.00 (52.74) | 3 |
|  |  | **Right** | 2.81 (3.64) | -88.71 (6.65) | -4.65 (2.50) | 290.33 (141.97) | 6 |
|  | **V5** | **Left** | -47.29 (6.86) | -68.96 (4.56) | 4.04 (6.41) | 585.29 (679.84) | 7 |
|  |  | **Right** | 45.26 (7.32) | -65.39 (5.60) | 4.29 (3.84) | 388.62 (390.03) | 8 |
| **NewPro** | **V1** | **Left** | -6.89 (2.70) | -91.89 (1.12) | -1.76 (4.71) | 470.00 (30.75) | 4 |
|  |  | **Right** | 6.62 (5.11) | -89.33 (8.06) | 0.68 (4.89) | 291.80 (64.09) | 5 |
|  | **V5** | **Left** | -44.46 (5.71) | -72.13 (5.83) | 6.46 (2.46) | 1005.12 (1052.05) | 8 |
|  |  | **Right** | 45.86 (4.17) | -67.88 (5.21) | 3.69 (4.53) | 824.44 (825.58) | 9 |

Coordinates (x, y and z) are in MNI space. Voxel number indicates the number of voxels constituting each ROI. Participants (n) denotes the number of subjects contributing to each mean.

**Table S4.** Adjusted means of regional cortical areas (mm^2^) and regional cortical thicknesses (mm) of children.

|  | OldPro Group (n=38) | NewPro Group (n=47) | p_value | Corrected p (FDR) |
| --- | --- | --- | --- | --- |
| Medial occipital area | 9601.93 ± 202.98 | 9176.80 ± 182.12 | 0.127 | 0.237 |
| Lateral occipital area | 12318.65 ± 285.48 | 12386.22 ± 256.14 | 0.862 | 0.862 |
| Medial parietal area | 15075.43 ± 301.16 | 14731.80 ± 270.21 | 0.403 | 0.489 |
| Lateral parietal area | 22139.60 ± 419.32 | 23239.84 ± 376.23 | 0.057 | 0.152 |
| Medial occipital thickness | 4.67 ± 0.06 | 4.61 ± 0.05 | 0.428 | 0.489 |
| Lateral occipital thickness | 2.59 ± 0.03 | 2.39 ± 0.02 | <0.001* | <0.001* |
| Medial parietal thickness | 8.49 ± 0.08 | 8.33 ± 0.07 | 0.148 | 0.237 |
| Lateral parietal thickness | 5.75 ± 0.06 | 5.46 ± 0.05 | 0.001* | 0.004* |

Data are presented as adjusted mean± standard error. The means have been adjusted for socioeconomic status.

**Table S5.** Multiple linear regression analyses predicting BOLD percent signal change (log-transformed) as a function of occipital area or parietal area.

| **Predictor Variable** | **Brain Area** | **RDK Coherence** | **β (95% CI)** | **t** | **p** | **Corrected p (FDR)** | **R-squared** | **Adjusted R-squared** |
| --- | --- | --- | --- | --- | --- | --- | --- | --- |
| **Occipital Area** | **V1** | **0%** | -0.620 (-1.511, 0.271) | -1.645 | 0.144 | 0.322 | 0.428 | -0.062 |
|  |  | **100%** | -0.677 (-1.565, 0.211) | -1.802 | 0.114 | 0.322 | 0.432 | -0.055 |
|  | **V5** | **0%** | 0.028 (-0.631, 0.688) | 0.092 | 0.928 | 0.928 | 0.367 | 0.095 |
|  |  | **100%** | -0.268 (-0.999, 0.463) | -0.787 | 0.444 | 0.593 | 0.222 | -0.111 |
| **Parietal Area** | **V1** | **0%** | 1.287 (-0.127, 2.700) | 2.153 | 0.068 | 0.322 | 0.523 | 0.114 |
|  |  | **100%** | 0.777 (-0.955, 2.508) | 1.061 | 0.324 | 0.519 | 0.284 | -0.331 |
|  | **V5** | **0%** | 0.791 (-0.356, 1.938) | 1.479 | 0.161 | 0.322 | 0.452 | 0.217 |
|  |  | **100%** | 0.327 (-1.057, 1.711) | 0.507 | 0.620 | 0.709 | 0.203 | -0.139 |

BOLD percent signal change was measured in V1 and V5, in response to RDK coherences of 0% and 100%. Associations were adjusted for cortical area, birth weight (z-scored), sex, socioeconomic status and group. Standardized coefficients (β), standardized confidence intervals, t-values, p-values, corrected p-values, r-squared values and adjusted r-squared values are reported.

**Table S6.** Multiple linear regression analyses predicting motion coherence threshold (log-transformed).

| **Predictor Variable** | **β (95% CI)** | **t** | **p** | **Corrected p (FDR)** | **R-squared** | **Adjusted R-squared** |
| --- | --- | --- | --- | --- | --- | --- |
| **Occipital Area** | 0.116 (-0.283, 0.515) | 0.579 | 0.564 | 0.752 | 0.091 | 0.046 |
| **Parietal Area** | 0.157 (-0.350, 0.664) | 0.615 | 0.540 | 0.752 | 0.092 | 0.047 |
| **Occipital Thickness** | -0.143 (-0.520, 0.234) | -0.754 | 0.453 | 0.752 | 0.093 | 0.047 |
| **Parietal Thickness** | 0.026 (-0.669, 0.720) | 0.073 | 0.942 | 0.942 | 0.086 | 0.041 |

Results are shown for four different predictors: occipital area and parietal area (adjusted for cortical area, socioeconomic status and group), and occipital thickness and parietal thickness (adjusted for mean cortical thickness, socioeconomic status and group). Standardized coefficients (β), standardized confidence intervals, t-values, p-values, corrected p-values, r-squared values and adjusted r-squared values are reported.

**Table S7.** Group interaction effects for multiple linear regression analyses predicting motion coherence threshold (log-transformed).

| **Interaction Term** | **Coef.** | **Std.Err.** | **t** | **P>\|t\|** | **Corrected p (FDR)** | **[0.025** | **0.975]** |
| --- | --- | --- | --- | --- | --- | --- | --- |
| **Group x Occipital Area** | 9.14e-06 | 4.17e-05 | 0.219 | 0.827 | 0.827 | -7.39e-05 | 9.22e-05 |
| **Group x Parietal Area** | 2.08e-05 | 2.70e-05 | 0.772 | 0.442 | 0.590 | -3.29e-05 | 7.46e-05 |
| **Group x Occipital Thickness** | -0.989 | 0.456 | -2.167 | 0.033 | 0.076 | -1.898 | -0.081 |
| **Group x Parietal Thickness** | -0.569 | 0.269 | -2.113 | 0.038 | 0.076 | -1.105 | -0.033 |

Results are shown for four different predictors: occipital area and parietal area (adjusted for cortical area and socioeconomic status), and occipital thickness and parietal thickness (adjusted for mean cortical thickness and socioeconomic status). Coefficients, standard errors, t-values, p-values, corrected p-values, and 95% confidence intervals are reported.

**Table S8.** Group interaction effects for multiple linear regression analyses predicting BOLD percent signal change (log-transformed) as a function of occipital area or parietal area.

| **Interaction Term** | **Area** | **RDK Coherence** | **Coef.** | **Std.Err.** | **t** | **P>\|t\|** | **Corrected p (FDR)** | **[0.025** | **0.975]** |
| --- | --- | --- | --- | --- | --- | --- | --- | --- | --- |
| **Group x Occipital Area** | **V1** | **0%** | 1.66e-04 | 2.62e-04 | 0.634 | 0.549 | 0.853 | -4.75e-04 | 8.07e-04 |
|  |  | **100%** | 4.11e-04 | 2.98e-04 | 1.380 | 0.217 | 0.853 | -3.18e-04 | 0.001 |
|  | **V5** | **0%** | -6.29e-05 | 1.58e-04 | -0.398 | 0.697 | 0.853 | -4.05e-04 | 2.79e-04 |
|  |  | **100%** | -5.74e-05 | 1.58e-04 | -0.363 | 0.723 | 0.853 | -3.99e-04 | 2.85e-04 |
| **Group x Parietal Area** | **V1** | **0%** | 8.57e-05 | 1.95e-04 | 0.440 | 0.675 | 0.853 | -3.91e-04 | 5.62e-04 |
|  |  | **100%** | -2.03e-05 | 3.07e-04 | -0.066 | 0.949 | 0.949 | -7.72e-04 | 7.31e-04 |
|  | **V5** | **0%** | 4.55e-05 | 1.09e-04 | 0.417 | 0.683 | 0.853 | -1.90e-04 | 2.81e-04 |
|  |  | **100%** | -3.93e-05 | 1.19e-04 | -0.330 | 0.747 | 0.853 | -2.96e-04 | 2.18e-04 |

BOLD percent signal change was measured in V1 and V5, in response to RDK coherences of 0% and 100%. Associations were adjusted for cortical area, birth weight (z-scored), sex, and socioeconomic status. Coefficients, standard errors, t-values, p-values, corrected p-values, and 95% confidence intervals are reported.

**Table S9.** Group interaction effects for multiple linear regression analyses predicting BOLD percent signal change (log-transformed) as a function of occipital thickness or parietal thickness.

| **Interaction Term** | **Area** | **RDK Coherence** | **Coef.** | **Std.Err.** | **t** | **P>\|t\|** | **Corrected p (FDR)** | **[0.025** | **0.975]** |
| --- | --- | --- | --- | --- | --- | --- | --- | --- | --- |
| **Group x Occipital Thickness** | **V1** | **0%** | -0.964 | 1.598 | -0.603 | 0.568 | 0.891 | -4.875 | 2.946 |
|  |  | **100%** | 0.246 | 1.724 | 0.142 | 0.891 | 0.891 | -3.974 | 4.465 |
|  | **V5** | **0%** | -4.106 | 1.588 | -2.586 | 0.023 | 0.181 | -7.536 | -0.675 |
|  |  | **100%** | -1.848 | 1.377 | -1.343 | 0.202 | 0.405 | -4.823 | 1.126 |
| **Group x Parietal Thickness** | **V1** | **0%** | 0.436 | 1.428 | 0.305 | 0.771 | 0.891 | -3.059 | 3.931 |
|  |  | **100%** | 0.324 | 1.357 | 0.239 | 0.819 | 0.891 | -2.997 | 3.644 |
|  | **V5** | **0%** | -1.371 | 0.793 | -1.729 | 0.108 | 0.289 | -3.085 | 0.342 |
|  |  | **100%** | -1.033 | 0.599 | -1.724 | 0.108 | 0.289 | -2.327 | 0.261 |

BOLD percent signal change was measured in V1 and V5, in response to RDK coherences of 0% and 100%. Associations were adjusted for cortical thickness, birth weight (z-scored), sex and socioeconomic status. Coefficients, standard errors, t-values, p-values, corrected p-values, and 95% confidence intervals are reported.

**Table S10.** Multiple linear regression analyses predicting BOLD percent signal change (log-transformed) as a function of temporal thickness.

| **Predictor Variable** | **Brain Area** | **RDK Coherence** | **β (95% CI)** | **t** | **p** | **Corrected p (FDR)** | **R-squared** | **Adjusted R-squared** |
| --- | --- | --- | --- | --- | --- | --- | --- | --- |
| **Temporal Thickness** | **V1** | **0%** | -0.540 (-2.179, 1.098) | -0.780 | 0.461 | 0.717 | 0.270 | -0.356 |
|  |  | **100%** | 0.475 (-1.260, 2.210) | 0.648 | 0.538 | 0.717 | 0.182 | -0.520 |
|  | **V5** | **0%** | 0.114 (-1.330, 1.558) | 0.169 | 0.868 | 0.868 | 0.302 | 0.003 |
|  |  | **100%** | 1.390 (0.107, 2.673) | 2.323 | 0.036 | 0.143 | 0.449 | 0.213 |

BOLD percent signal change was measured in V1 and V5, in response to RDK coherences of 0% and 100%. Associations were adjusted for mean cortical thickness, birth weight (z-scored), sex, socioeconomic status and group. Standardized coefficients (β), standardized confidence intervals, t-values, p-values, corrected p-values, r-squared values and adjusted r-squared values are reported.

**Table S11.** Group interaction effects for multiple linear regression analyses predicting BOLD percent signal change (log-transformed) as a function of lateral occipital thickness or lateral parietal thickness.

| **Interaction Term** | **Area** | **RDK Coherence** | **Coef.** | **Std.Err.** | **t** | **P>\|t\|** | **Corrected p (FDR)** | **[0.025** | **0.975]** |
| --- | --- | --- | --- | --- | --- | --- | --- | --- | --- |
| **Group x Lateral Occipital Thickness** | **V1** | **0%** | -0.340 | 3.167 | -0.107 | 0.918 | 0.918 | -8.088 | 7.409 |
|  |  | **100%** | 2.219 | 3.452 | 0.643 | 0.544 | 0.622 | -6.226 | 10.665 |
|  | **V5** | **0%** | -4.387 | 1.714 | -2.559 | 0.024 | 0.190 | -8.090 | -0.683 |
|  |  | **100%** | -1.619 | 1.767 | -0.917 | 0.376 | 0.605 | -5.436 | 2.197 |
| **Group x Lateral Parietal Thickness** | **V1** | **0%** | 1.381 | 1.715 | 0.805 | 0.452 | 0.605 | -2.816 | 5.578 |
|  |  | **100%** | 1.578 | 1.971 | 0.801 | 0.454 | 0.605 | -3.244 | 6.400 |
|  | **V5** | **0%** | -1.531 | 0.917 | -1.670 | 0.119 | 0.475 | -3.512 | 0.449 |
|  |  | **100%** | -1.008 | 0.763 | -1.320 | 0.210 | 0.559 | -2.657 | 0.642 |

BOLD percent signal change was measured in V1 and V5, in response to RDK coherences of 0% and 100%. Associations were adjusted for mean cortical thickness, birth weight (z-scored), sex and socioeconomic status. Coefficients, standard errors, t-values, p-values, corrected p-values, and 95% confidence intervals are reported.

**Table S12.** Multiple linear regression analyses predicting BOLD percent signal change (log-transformed) as a function of lateral occipital thickness or lateral parietal thickness.

| **Predictor Variable** | **Brain Area** | **RDK Coherence** | **β (95% CI)** | **t** | **p** | **Corrected p (FDR)** | **R-squared** | **Adjusted R-squared** |
| --- | --- | --- | --- | --- | --- | --- | --- | --- |
| **Lateral Occipital Thickness** | **V1** | **0%** | -0.894 (-2.302, 0.514) | -1.502 | 0.177 | 0.283 | 0.400 | -0.114 |
|  |  | **100%** | -1.275 (-2.526, -0.024) | -2.411 | 0.047* | 0.187 | 0.526 | 0.120 |
|  | **V5** | **0%** | -0.047 (-1.090, 0.996) | -0.097 | 0.924 | 0.924 | 0.301 | 0.001 |
|  |  | **100%** | -0.746 (-1.749, 0.257) | -1.595 | 0.133 | 0.283 | 0.354 | 0.077 |
| **Lateral Parietal Thickness** | **V1** | **0%** | -0.163 (-1.843, 1.517) | -0.230 | 0.825 | 0.924 | 0.212 | -0.463 |
|  |  | **100%** | -0.994 (-2.517, 0.528) | -1.544 | 0.167 | 0.283 | 0.353 | -0.202 |
|  | **V5** | **0%** | -0.222 (-1.387, 0.942) | -0.410 | 0.688 | 0.918 | 0.309 | 0.012 |
|  |  | **100%** | -1.128 (-2.167, -0.089) | -2.330 | 0.035* | 0.187 | 0.450 | 0.214 |

BOLD percent signal change was measured in V1 and V5, in response to RDK coherences of 0% and 100%. Associations were adjusted for mean cortical thickness, birth weight (z-scored), sex, socioeconomic status and group. Standardized coefficients (β), standardized confidence intervals, t-values, p-values, corrected p-values, r-squared values and adjusted r-squared values are reported.
