# Supplementary figures and images for: Parietal thickness predicts middle temporal area (V5) motion responses in 7-year-old children born very preterm
Source: Cereb Cortex. 2026 Jun 24;36(6):bhag089. doi: 10.1093/cercor/bhag089 (PMC13293252; doi:10.1093/cercor/bhag089)

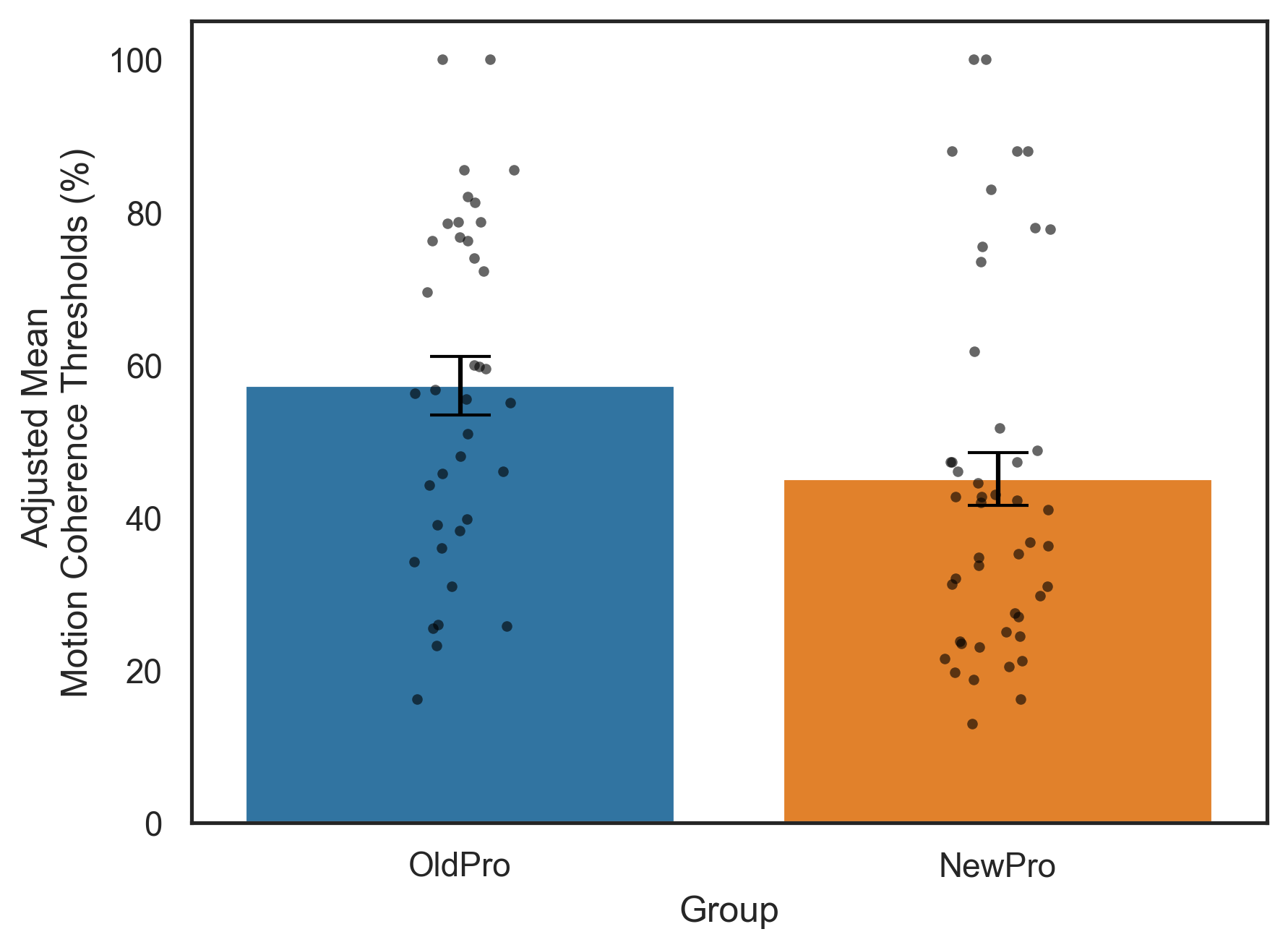

Supplement: Supplementary_material_bhag089 [file supplementary_material_bhag089.zip › FIG_S3.png]

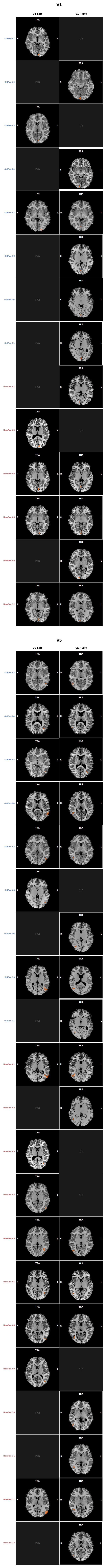

Supplement: Supplementary_material_bhag089 [file supplementary_material_bhag089.zip › FIG_S1.png]

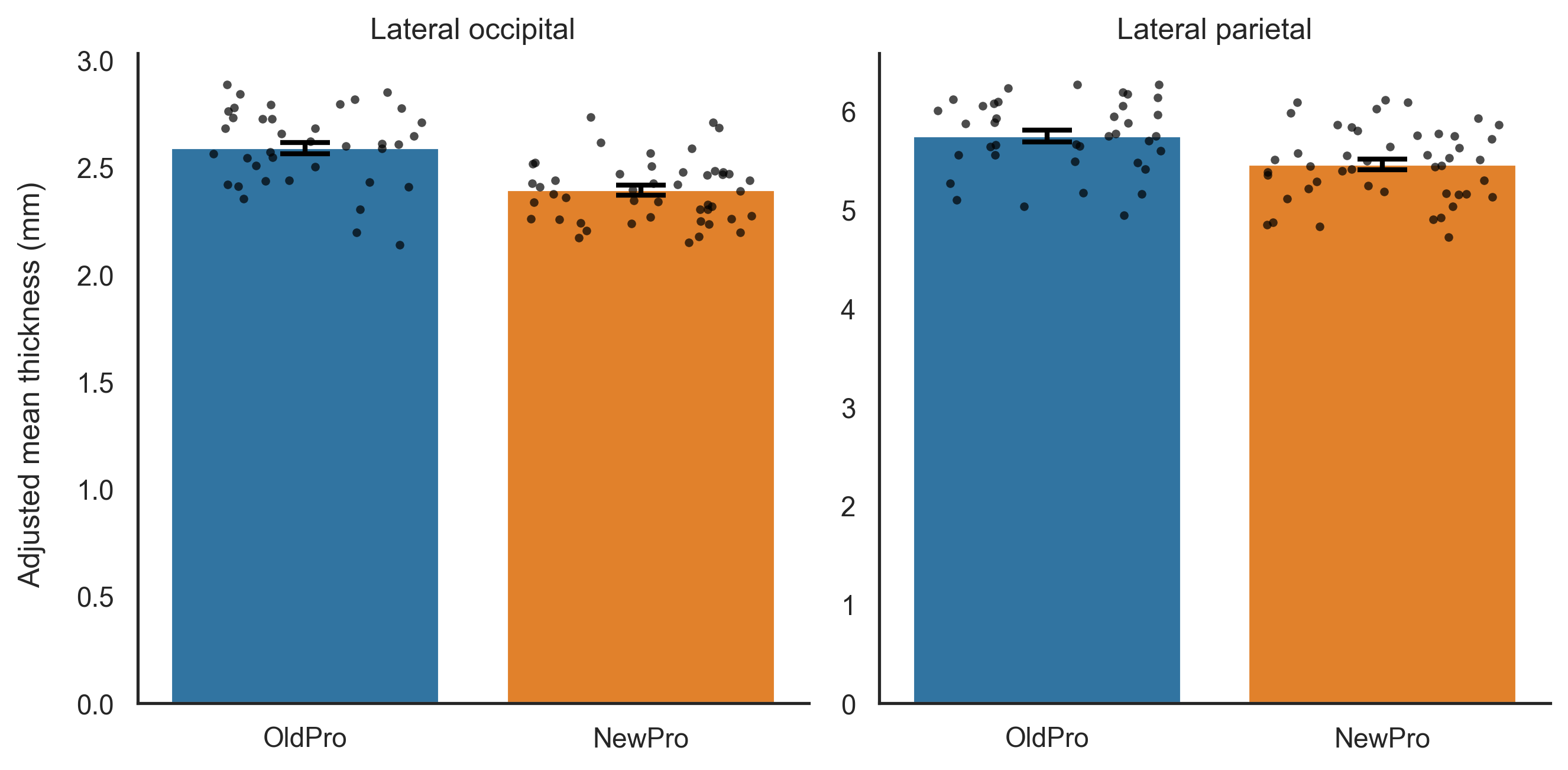

Supplement: Supplementary_material_bhag089 [file supplementary_material_bhag089.zip › FIG_S2.png]
